# Dehydrocrenatidine Inhibits Voltage-Gated Sodium Channels and Ameliorates Mechanic Allodia in a Rat Model of Neuropathic Pain

**DOI:** 10.3390/toxins11040229

**Published:** 2019-04-18

**Authors:** Fang Zhao, Qinglian Tang, Jian Xu, Shuangyan Wang, Shaoheng Li, Xiaohan Zou, Zhengyu Cao

**Affiliations:** Jiangsu Key Laboratory of TCM Evaluation and Translational Research, School of Traditional Chinese Pharmacy, China Pharmaceutical University, Nanjing 211198, China; 1620184499@cpu.edu.cn (F.Z.); cputangqinglian@163.com (Q.T.); xujian0118@163.com (J.X.); shuangyanwcpu@163.com (S.W.); lsh199242@163.com (S.L.)

**Keywords:** dehydrocrenatidine, neuropathic pain, voltage-gated sodium channels

## Abstract

*Picrasma quassioides* (D. Don) Benn, a medical plant, is used in clinic to treat inflammation, pain, sore throat, and eczema. The alkaloids are the main active components in *P. quassioides*. In this study, we examined the analgesic effect of dehydrocrenatidine (DHCT), a β-carboline alkaloid abundantly found in *P. quassioides* in a neuropathic pain rat model of a sciatic nerve chronic constriction injury. DHCT dose-dependently attenuated the mechanic allodynia. In acutely isolated dorsal root ganglion, DHCT completely suppressed the action potential firing. Further electrophysiological characterization demonstrated that DHCT suppressed both tetrodotoxin-resistant (TTX-R) and sensitive (TTX-S) voltage-gated sodium channel (VGSC) currents with IC_50_ values of 12.36 μM and 4.87 µM, respectively. DHCT shifted half-maximal voltage (V_1/2_) of inactivation to hyperpolarizing direction by ~16.7 mV in TTX-S VGSCs. In TTX-R VGSCs, DHCT shifted V_1/2_ of inactivation voltage to hyperpolarizing direction and V_1/2_ of activation voltage to more depolarizing potential by ~23.9 mV and ~12.2 mV, respectively. DHCT preferred to interact with an inactivated state of VGSCs and prolonged the repriming time in both TTX-S and TTX-R VGSCs, transiting the channels into a slow inactivated state from a fast inactivated state. Considered together, these data demonstrated that the analgesic effect of DHCT was likely though the inhibition of neuronal excitability.

## 1. Introduction

Neuropathic pain affects 6–8% of the population, among which 27% of patients are in a condition of chronic pain [1]. Despite the existence of seven categories of pain drugs, only 30% of patients get adequate relief. In addition, currently used analgesics produce significant side effects, such as addiction and sedation, which negatively affect life quality of the patients [2].

Neuropathic pain results from disorders of peripheral and/or central nervous systems. The pathological mechanisms are complex. Sensitization of neurons leads to enhanced neuronal excitability therefore representing the major cause of abnormal nociception [3]. Alterations in the activities and/or expression levels of ion channels, such as calcium, sodium, and potassium channels, have been demonstrated in neurons from neuropathic pain rodent models and contribute to the hyper-excitability of injured neurons [4,5,6,7]. Voltage-gated sodium channels (VGSCs) are responsible for the rising phase of action potential (AP) generation, therefore controlling neuronal excitability [8,9,10]. The altered expression levels and sensitivities of VGSCs in dorsal root ganglion (DRG) neurons in painful neuropathy result in hyper-excitability of neurons [11,12,13].

*Picrasma quassioides* (D. Don) Benn, a medical plant, has been used in clinics to treat diseases, such as inflammation, pain, sore throat, gastroenteritis, and eczema [14,15]. Phytochemical investigations revealed the presence of many types of chemical constituents such as alkaloids (mainly β-carboline and cathinone alkaloids), bitter principles (nigakihemiacetal A–F, nigakilactone A–N, picrasin C–G, picraqualides A–D and picrasinoside A–G), and triterpenoids in this medical plant [14,16,17,18,19]. Pharmacological studies demonstrate that the ethanolic extract of *P. quassioides* stems displays anti-cancer, anti-diabetes, and anti-hypertensive activities [14,17,20]. The total alkaloids extracted from *P. quassioides* are the main active components for antipyretic, anti-inflammatory, anti-bacterial, and anti-tumor activities [20,21]. In addition, these alkaloids are also effective in the peripheral nervous system and central nervous system. The total alkaloid fraction suppresses the sympathetic nerve firing therefore displaying an anti-hypertension effect [22].

Dehydrocrenatidine (DHCT) is one of the most abundant β-carboline alkaloids found in the *P. quassioides*. The levels of DHCT in the stems of *P. quassiodes* can reach as high as 2.72% [23]. DHCT has been reported to suppress lipopolysaccharide (LPS)-stimulated NO production and secretion of pro-inflammatory cytokines including tumor necrosis factor α (TNF-α) and interleukin-6 (IL-6) through inhibition of inducible nitric oxide synthase (iNOS) and cyclooxygenase-2 (COX-2) activities, as well as the expression levels in macrophages [15]. Recently, a study has also demonstrated that DHCT is a specific janus kinase (JAK)-specific inhibitor that induces DU145 and MDA-MB-468 cell apoptosis [24]. In addition, several synthesized β-carboline alkaloids possess anxiolytic and anticonvulsant, sedative effects through interaction with 5-hydroxytryptamine, dopamine, and benzodiazepine receptors suggesting their effects on neuronal excitability [25,26,27,28,29]. However, whether and how DHCT is able to affect neuronal excitability has never been explored, despite its high concentration in *P. quassioides,* which is used for treatment of pain in the clinic.

In the current study, we evaluated the analgesic activity of DHCT, a representative of β-carboline alkaloid from *P. quassioides*, in a rat neuropathic pain model. We demonstrate that DHCT ameliorates neuropathic pain. Mechanistically, DHCT suppresses VGSC Na^+^ currents and AP firing in DRG neurons.

## 2. Results

### 2.1. Dehydrocrenatidine Ameliorated Mechanic Allodynia in a Neuropathic Pain Model of Sciatic Nerve Partial Ligation

**Figure 1:**
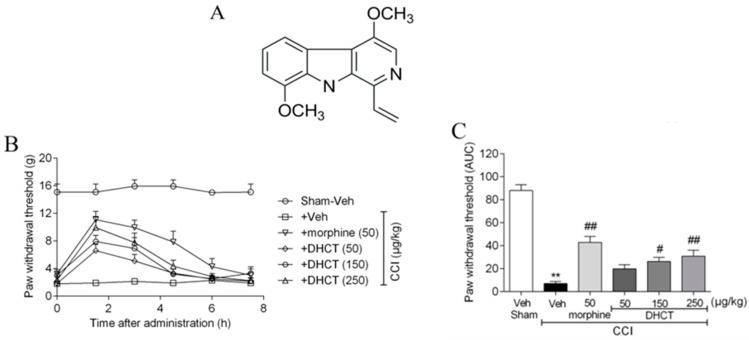
Dehydrocrenatidine (DHCT) attenuated sciatic nerve partial ligation-induced mechanical allodynia. (**A**) Chemical structure of DHCT. (**B**) Mechanical stimuli thresholds recorded after administration of vehicle (5% DMSO, 5% Tween-80, and 90% normal saline), DHCT (50, 150, and 250 μg/kg) or morphine (50 μg/kg) every 1.5 h, respectively. (**C**) Quantification of DHCT analgesic effects by using area under the curve (the area of paw withdrawal thresholds during 0–4.5 h after intrathecal administration). DHCT displayed significant analgesic effects in the rat chronic constriction neuropathic pain model. Each data point represents the mean ± SEM (*n* = 9). ** *p* < 0.01, model vs. sham group; # *p* < 0.05; ## *p* < 0.01, drugs vs. model group.

As a representative of β-carboline alkaloid in *P. quassioides*, the analgesic effect of DHCT (Figure 1A) was investigated in a chronic constrictive injury (CCI) neuropathic pain model. Consistent with previous reports, after ligation of the sciatic nerve for 14 days, the paw withdrawal threshold (PWT) in CCI rats reached 1.90 ± 0.31 g, which was significantly lower than that observed in the sham group (15.10 ± 1.50 g, *n* = 9, *p* < 0.01) (Figure 1B). Intrathecal (*i.t.*) administration of DHCT (150 or 250 μg/kg) significantly attenuated CCI-induced mechanical allodynia (Figure 1B,C). The analgesic effect was peaked at 1.5 h post administration and lasted at least for 3 h (Figure 1B). At the highest dose (250 µg/kg) investigated, DHCT produced comparable efficacy with the positive control, morphine (50 μg/kg) (Figure 1B).

### 2.2. DHCT Suppressed Action Potential Generation in Acutely Dissociated Rat DRG Neurons

**Figure 2:**
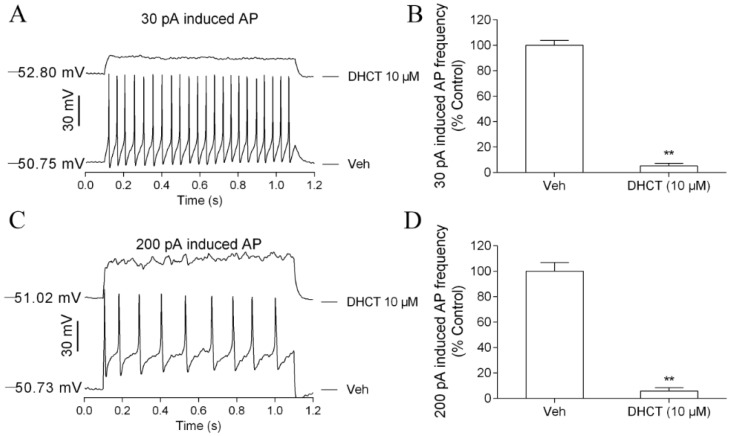
DHCT suppressed current-evoked action potential in dorsal root ganglion (DRG) neurons. (**A**) Representative traces of action potential (APs) evoked by an injection of a 30-pA (1 s) current in the absence and presence of 10 µM of DHCT. (**B**) Quantification of DHCT (10 μM) suppressed 30-pA induced APs in acutely dissociated rat DRG neurons. (**C**) Representative traces of APs evoked by an injection of a 200-pA (1 s) current in the absence and presence of 10 µM of DHCT. (**D**) Quantification of DHCT (10 μM) suppressed 200-pA induced APs in acutely dissociated rat DRG neurons. Each data point represents mean ± SEM. T-test was used to compared the statistical significance between Veh (0.1% DMSO) and DHCT (10 µM) groups. ** *p* < 0.01, *n* = 13.

Given the analgesic effect of DHCT on CCI neuropathic pain model, we next examined whether DHCT was capable of suppressing neuronal excitability. Injection of a 30-pA (1 s) or 200-pA (1 s) current evoked repetitive APs (Figure 2A–C lower trace) in small or medium diameter DRG neurons. Bath application of DHCT (10 µM) for 1 min before current injections suppressed the firing frequency of APs elicited by injections of 30-pA and 200-pA currents in DRG neurons (Figure 2).

### 2.3. DHCT Suppressed both Tetrodotoxin-Sensitive (TTX-S) and TTX-Resistant (TTX-R) VGSC Na^+^ Currents in DRG Neurons

**Figure 3:**
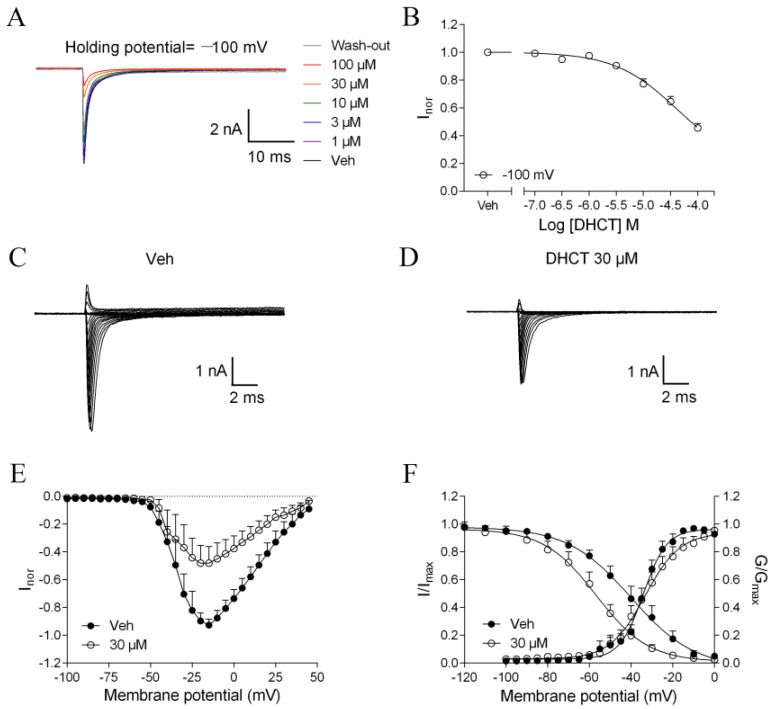
DHCT suppressed tetrodotoxin-sensitive (TTX-S) Na^+^ currents in medium- and large-diameter -diameter DRG neurons. (**A**) Representative traces of DHCT suppressing TTX-S Na^+^ currents in medium- and large-diameter DRG neurons. (**B**) Concentration–response curve of DHCT suppressed TTX-S Na^+^ currents. Sodium currents were evoked by a 50-ms depolarization pulse to 0 mV from a holding potential of −100 mV. (**C**,**D**) Representative TTX-S Na^+^ currents evoked by different depolarizing voltages from −90 to +50 mV in a 5-mV step in the absence (C) and presence (D) of DHCT (30 µM). (**E**) Current–voltage (I-V) relationships of TTX-S VGSCs in the presence or absence of DHCT. (**F**) Steady-state activation and inactivation curves of TTX-S Na^+^ currents in the absence and presence of DHCT (30 µM). Each data point represents mean ± SEM (*n* = 5).

**Figure 4:**
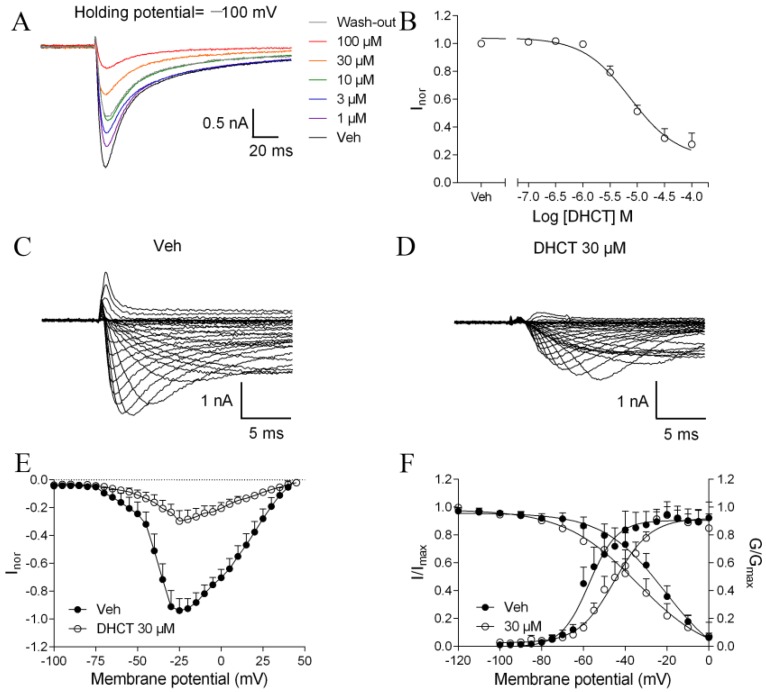
DHCT suppressed tetrodotoxin-resistant (TTX-R) Na^+^ currents in small-diameter DRG neurons in the presence of 300 nM TTX. (**A**) Representative traces of DHCT suppressing TTX-R Na^+^ currents. (**B**) Concentration–response curve of DHCT suppressed TTX-R Na^+^ currents. Sodium currents were evoked by a 50-ms depolarization pulse to 0 mV from a holding potential of −100 mV. (**C**,**D**) Representative TTX-R Na^+^ currents evoked by different depolarizing voltages from −90 to +50 mV in a 5-mV step in the absence (**C**) and presence (**D**) of DHCT (30 µM). (**E**) Current–voltage (I-V) relationships of TTX-R VGSCs in the presence or absence of 30 µM DHCT. (**F**) Steady-state activation and inactivation curves of TTX-R Na^+^ currents in the absence and presence of DHCT (30 µM). Each data point depicts mean ± SEM (*n* = 5).

Given the inhibitory effect on AP generation in acutely dissociated rat DRG neurons and the pivotal role of VGSCs on neuroexcitability, we next examined the activity of DHCT on VGSCs in DRG neurons. Sodium currents were evoked by a 50-ms depolarization pulse to 0 mV from a holding potential of −100 mV. In medium- and large-diameter DRG neurons, membrane depolarization induced fast activated and fast inactivated Na^+^ currents consistent with TTX-S VGSC currents [30]. Tetrodotoxin (TTX) (0.1–10 nM) concentration-dependently suppressed VGSC currents (Figure S1A,B). At a concentration of 10 nM, TTX produced a nearly complete suppression on VGSC Na^+^ currents suggesting that medium- and large-diameter DRG neurons mainly express TTX-S VGSCs (Figure S1). DHCT concentration-dependently suppressed the Na^+^ current in medium- and large-diameter DRG neurons with an IC_50_ value of 23.47 μM (14.59–37.74 μM, 95% confidential interval (CI)) (Figure 3A,B). To examine the voltage-dependence of DHCT action in TTX-S VGSCs, Na^+^ currents were elicited by depolarization steps from −90 to +50 mV in a 5-mV increment in the presence or absence of DHCT (30 µM). DHCT suppressed the Na^+^ peak currents evoked by different depolarization potentials (Figure 3C,D). The current–voltage (I-V) relationships in the presence and absence of 30 μM DHCT were illustrated in Figure 3E. Bath application of DHCT (30 µM) dramatically shifted the half-maximal voltage (V_1/2_) of steady-state inactivation from −40.07 mV to −56.73 mV, but not activation V_1/2_ of TTX-S VGSCs (Figure 3F). In small-diameter DRG neurons, in the presence of 300 nM TTX, depolarization induced slow activated and slow inactivated Na^+^ currents (Figure 4A). Bath application of DHCT (30 µM) suppressed TTX-R Na^+^ currents with an IC_50_ value of 7.42 μM (4.91–11.22 μM, 95% CI) (Figure 4A,B). DHCT (30 µM) suppressed the TTX-R Na^+^ peak currents evoked by different depolarization potentials (Figure 4C–E). DHCT shifted the V_1/2_ for activation of TTX-R VGSCs from −57.32 mV to −45.08 mV and V_1/2_ for inactivation from −21.22 mV to −34.08 mV (Figure 4F).

### 2.4. DHCT Preferred to Interact with Inactivated State of VGSCs

**Figure 5:**
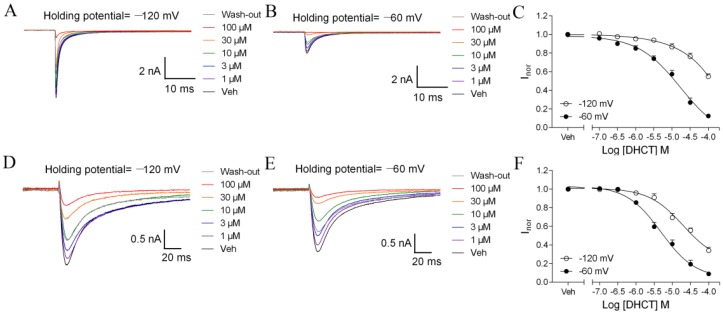
DHCT preferentially interacted with an inactivated state of TTX-S and TTX-R VGSCs in DRG neurons. (**A**) Representative TTX-S Na^+^ current traces in the absence and presence of different concentrations of DHCT evoked by a depolarization potential to 0 mV from a holding potential of −120 mV. (**B**) Representative TTX-S Na^+^ current traces in the absence and presence of different concentrations of DHCT evoked by a depolarization potential to 0 mV from a holding potential of −60 mV. (**C**) Concentration–response curves of DHCT suppressing TTX-S Na^+^ currents at the holding potentials of −120 mV and −60 mV, respectively. (**D**) Representative TTX-R Na^+^ current traces in the absence and presence of different concentrations of DHCT evoked by a depolarization potential to 0 mV from a holding potential of −120 mV. (**E**) Representative TTX-R Na^+^ current traces in the absence and presence of different concentrations of DHCT evoked by a depolarization potential to 0 mV from a holding potential of −60 mV. (**F**) Concentration–response curves of DHCT suppressing TTX-R Na^+^ currents in DRG neurons at the holding potentials of −120 mV and −60 mV, respectively. Each data point represents mean ± SEM (*n* = 5–9).

Many VGSC gating modifiers affect VGSC gating by preferentially interacting with an inactivated state of VGSCs [31,32,33]. Given the robust shift on V_1/2_ for inactivation of DHCT on both TTX-S and TTX-R VGSCs, we next investigated whether DHCT preferentially interacted with inactivated state of VGSCs. When Na^+^ currents were elicited by a depolarizing voltage step to 0 mV from −120 mV (950 ms), at which channels were predominantly in a resting state, DHCT concentration-dependently suppressed the TTX-S Na^+^ currents in medium- and large-diameter DRG neurons with an IC_50_ value of 57.25 μM (27.31–120.00 μM, 95% CI) (Figure 5A–C). When a test pulse depolarized to 0 mV from a 950-ms conditioning voltage of −60 mV, at which, approximately 60% of TTX-S sodium channels were in an inactivated state, DHCT suppressed the TTX-S Na^+^ currents with an IC_50_ value of 12.36 μM (8.91–17.15 μM, 95% CI) (Figure 5B,C). Similarly, DHCT also suppressed the TTX-R Na^+^ currents in small-diameter DRG neurons in the presence of 300 nM TTX. The IC_50_ values were 19.97 μM (8.69–45.88 μM, 95% CI) and 4.87 μM (3.42–6.94 μM, 95% CI) when the holding potentials were −120 mV and −60 mV (~20% channels are in activated state), respectively (Figure 5D–F). The potency differences between distinct holding potentials suggested that DHCT preferentially bound to an inactivated state of VGSCs. The inactivated state dependence of DHCT was consistent with the hyperpolarized shifts of steady-state inactivation in both TTX-S and TTX-R VGSCs.

**Figure 6:**
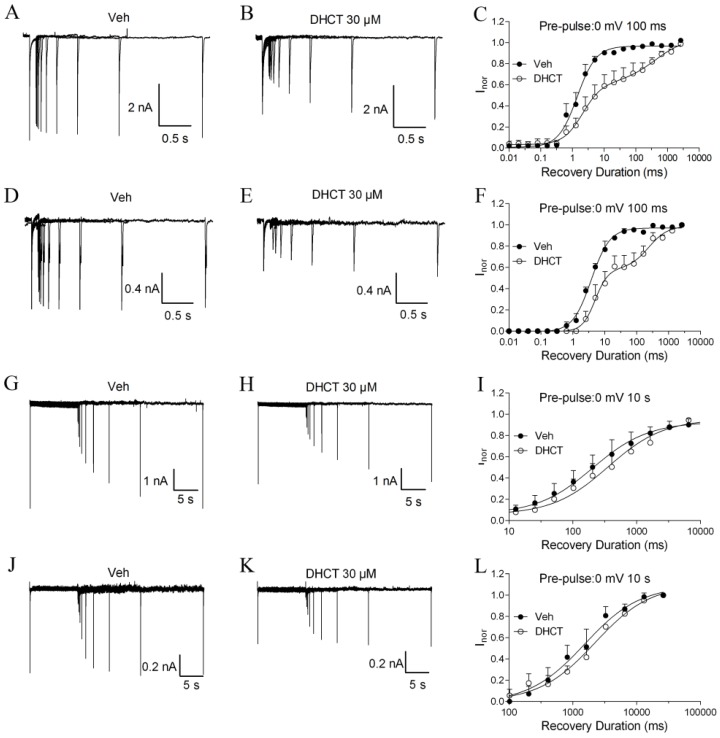
DHCT exposure transited both TTX-S and TTX-R Na^+^ currents from a fast-inactivated state to a slow-inactivated state. (**A**,**B**) Representative traces of TTX-S Na^+^ currents when the holding potential was set at 0 mV for 100 ms, followed by a recovery interval of variable duration to −120 mV before testing for an active current with a 20-ms pulse to 0 mV in the absence (**A**) and presence (**B**) of DHCT (30 µM). (**C**) Recovery curves of TTX-S Na^+^ currents from fast-inactivation in the absence and presence of DHCT. (**D**,**E**) Representative traces of TTX-R Na^+^ currents when the holding potential was set at 0 mV for 100 ms, followed by a recovery interval of variable duration to −120 mV before testing for an active current with a 20-ms pulse to 0 mV in the absence (**D**) and presence (**E**) of DHCT (30 µM). (**F**) Recovery curves of TTX-R Na^+^ currents from fast-inactivation in the absence and presence of DHCT. (**G**,**H**) Representative traces of TTX-S Na^+^ currents when the cells were pre-conditioned at 0 mV for 10 s, followed by a recovery interval of variable duration to −120 mV before testing for an active current with a 20-ms pulse to 0 mV in the absence (**G**) and presence (**H**) of DHCT (30 µM). (**I**) Recovery curves of TTX-S Na^+^ currents from slow-inactivation in the absence and presence of DHCT. (**J**,**K**) Representative traces of TTX-R Na^+^ currents when the cells were pre-conditioned at 0 mV for 10 s, followed by a recovery interval of variable duration to −120 mV before testing for an active current with a 20-ms pulse to 0 mV in the absence (**J**) and presence (**K**) of DHCT (30 µM). (**L**) Recovery curves of TTX-R Na^+^ currents from slow-inactivation in the absence and presence of DHCT. Each data point represents mean ± SEM (*n* = 6–8).

DHCT appeared to prolong the repriming time from an inactivated state when DRG neurons were held at a holding potential of 0 mV for 100 ms or 10 s, followed by a variable recovery interval at −120 mV before eliciting a current with a 20-ms pulse to 0 mV. The normalized current was plotted versus the recovery duration to determine the time constant (τ) of recovery from an inactivated state using first-, or second-order exponential growth equations [34]. When the precondition holding potential was set to be 0 mV for 100 ms, the recovery of TTX-S Na^+^ currents displayed a monophasic profile and was best fit by a first-order exponential growth equation with a τ value of 1.43 ms (1.19–1.71 ms, 95% CI), which was consistent with a fast inactivation recovery. In the presence of DHCT (30 µM), recovery of Na^+^ currents displayed a biphasic response and was best fit by a second-order exponential growth equation with τ values of 2.11 ms (1.15–5.11 ms, 95% CI) and 410.20 ms (23.88–7046.93 ms, 95% CI), respectively (Figure 6A–C). Similarly, in small-diameter DRG neurons in the presence of 300 nM TTX, bath application of DHCT also shifted a monophasic profile with a τ value of 3.80 ms (3.42–4.23 ms, 95% CI) to a biphasic response with τ values of 5.03 ms (3.16–8.02 ms, 95% CI) and 237.68 ms (89.54–632.41 ms, 95% CI), respectively (Figure 6D–F) suggesting that DHCT exposure transited both TTX-S and TTX-R Na^+^ currents from a fast-inactivated state to a slow-inactivated state. When the precondition holding potential was set to be 0 mV for 10 s, recovery of TTX-S Na^+^ currents displayed monophasic profiles in the absence and presence of DHCT with τ values of 205.9 ms (137.8–307.7 ms, 95% CI, *n* = 6) and 358.8 ms (254.1–506.7 ms, 95% CI, *n* = 6), respectively (Figure 6G–I). Similarly, recovery of TTX-R Na^+^ currents in the absence and presence of DHCT both displayed monophasic profiles with τ values of 1524.05 ms (1106.62–2094.11 ms, 95% CI, *n* = 6) and 2218.20 ms (1774.19–2779.71 ms, 95% CI, *n* = 6), respectively (Figure 6J–L). The prolonged recovery time constants in the presence of DHCT suggested that DHCT was able to stabilize the inactivation state of VGSCs.

## 3. Discussion

Although the mechanisms of neuropathic pain are complex, sensitization of nociceptors has been proposed to be the major cause for abnormal nociception [3]. VGSCs are responsible for the rising phase of the APs [8,35]. Sensitization of VGSC through various mechanisms has been demonstrated in the nociceptors from neuropathic rodent models and proposed to contribute to the hyper-excitability of injured neurons [4,5,6,7]. Discovery of VGSC gating modifiers, especially those displaying selectivity in VGSC subtypes (Na_v_1.7 and Na_v_1.8) is of high interest for pain therapy [36,37].

In this study, we investigated the analgesic effect of DHCT, a representative of β-carboline alkaloids found in the *P. quassiodes*, which was used clinically to treat various diseases including inflammation and pain as a traditional Chinese medicine. We demonstrated that DHCT displayed comparable efficacy with morphine in attenuation of mechanical allodynia in a sciatic nerve partial ligation neuropathic pain model. We further demonstrated that DHCT suppressed the neuronal excitability and inhibited the VGSC (both TTX-R and TTX-S) currents in DRG neurons. Considered together, these data demonstrated that the analgesic effect of DHCT was possibly through inhibition of VGSCs, one of the major mechanisms underlining neuropathic pain onset and progression [38]. It should be noted that CCI-injured DRG neurons displayed altered expression levels of sodium channel subtypes, such as Na_v_1.3, Na_v_1.8, and Na_v_1.9 [39,40]. Whether DHCT is also capable of suppressing AP firing in CCI-injured neurons needs further examination. Recently, DHCT has been demonstrated to be a Jasus kinase (JAK) inhibitor [24]. Many studies have revealed that blockade of JAK/Signal transducers and activators of transcription (STAT) signaling pathways decreased microglia activation [41,42], reduced the release of cytokines [43,44,45], and attenuated both mechanical allodynia and thermal hyperalgesia in a spinal nerve ligation neuropathic pain model [46]. Therefore, the analgesic effect of DHCT was possibly through dual inhibitions of VGSCs and JAK-mediated microglia activation, two major mechanisms underlining in neuropathic pain onset and progression.

In DRG neurons, DHCT suppressed AP generation, suggesting that DHCT was able to inhibit the neuronal excitability. Various β-carboline alkaloids produced either a sedative, tremorgenic, anxiogenic, or convulsant effect by binding to benzodiazepine (BZ) receptors acting as full, partial agonists, antagonists, or inverse agonists [19,22,26,27,28]. Whether DHCT interacted with BZ receptors and contributed to the neuronal excitability is currently unknown. In the current study, we observed an inhibitory effect of DHCT on Na^+^ currents in cultured DRG neurons. DHCT suppressed TTX-S and TTX-R Na^+^ currents at −60 mV with an IC_50_ value of 12.36 µM and 4.87 µM respectively, in DRG neurons, suggesting that TTX-R VGSCs were more sensitive to DHCT exposure. DHCT at 10 µM completely suppressed the APs evoked by a 30-pA or 200-pA current injection. It should be mentioned that the resting membrane potential of DRG neuron was approximately −50 mV [47]. Considering that DHCT preferentially bound to inactivated state of VGSCs, the potency of DHCT to suppress VGSCs Na^+^ currents in DRG neurons was therefore comparable to that on inhibition of AP firing. Therefore, it was likely that suppression of VGSCs was responsible for DHCT action in neuronal excitability.

Inhibition of both TTX-S and TTX-R Na^+^ currents by DHCT in DRG neurons was state-dependent. The IC_50_ value for DHCT suppression of VGSC activity was around 5-fold (TTX-S) and 4-fold (TTX-R) more potent in an inactivated state (−60 mV holding potential) than that of a resting state (−120 mV holding potential). This state-dependence of DHCT action was further confirmed by prolonged repriming time of Na^+^ current recovery. DHCT produced a depolarization shift of V_1/2_ on inactivation in TTX-S VGSCs. In addition to the depolarized shifts of inactivation voltages, DHCT also robustly shifted voltage-dependent activation to hyperpolarized potentials in TTX-R VGSCs. The electrophysiological property of DHCT appeared to be distinct to the VGSC pore blocker, TTX [48]. Most of VGSC inhibitors such as lidocaine bound to the local anesthetic sites, which were comprised of amino acids of Ile/Phe/Met (IFM) (domain III–IV (DIII–DIV linker)), and the residues located at IS6, IIS6, and IVS6 segments [49,50]. Although both lidocaine and DHCT produced a hyperpolarized shift on voltage-dependent inactivation, lidocaine had no effect on voltage-dependent activation [51,52]. In addition, the kinetics of DHCT prolonged repriming time from fast-inactivated state of VGSCs was also distinct to that of lidocaine [34]. Considered together, these data suggested that DHCT binding sites on VGSCs were distinct to TTX, and were not identical to that of local anesthetics. In the current study, we are not able to clarify why DHCT distinctly affected the gating kinetics between TTX-S and TTX-R VGSCs. Gating modifiers produce conformational changes and the coupling between the different states of VGSCs. In general, activators binding to the voltage sensor (VS) of DII affect activation, while activators binding to the VS of DIV generally affect inactivation [53]. Whether DHCT also bound to VSs of both DII and DIV of TTX-R VGSCs requires further exploration.

In summary, we demonstrated that DHCT suppressed the mechanic allodia in a rat model of neuropathic pain. We also demonstrated that DHCT preferentially binds to the inactivated state of VGSCs and therefore suppresses the VGSC currents and neuronal excitability. Our data provided novel information on the mode of actions for DHCT, and to an extent, for β-carboline alkaloids on neuronal excitability.

## 4. Materials and Methods

### 4.1. Materials

DHCT was generously provided by Professor Dequan Yu (Chinese Academy of Medical Sciences and Peking Union Medical College, Beijing, China) and purified as described previously [54]. The purity was determined to be over 95% using HPLC. Penicillin, streptomycin, trypsin, soybean trypsin inhibitor, L-glutamine, fetal bovine serum (FBS), Dulbecco minimum essential medium (DMEM), and Neurobasal medium were obtained from Life Technology (Grand Island, NY, USA). Poly-D-lysine (PDL), deoxyribonuclease I, TTX, nerve growth factor (NGF), and all inorganic chemicals were purchased from Sigma-Aldrich (St. Louis, MO, USA).

### 4.2. Animal Care

Animal protocols were approved by Institutional Animal Care and Use Committee of China Pharmaceutical University (12100000466006834N, SYXK 2016-0011, 27 January 2016). Efforts were made to minimize animal suffering and reduce the number of experimental animals. Male Sprague-Dawley (SD) rats (200–220 g) were purchased from Qing-Long-Shan Laboratory Animal Center (Nanjing, Jiangsu, China). All the animals were maintained in a temperature-controlled (23 ± 2 °C) vivarium at a 12-h light/dark cycle provided with food and water ad libitum.

### 4.3. Acutely Dissected Rat Dorsal Root Ganglion Neurons

Acutely dissociated rat dorsal root ganglion neurons (DRG) were obtained from native male SD rats (200–220 g) as described previously [47,55]. In brief, the dissected DRG tissue was digested with 0.05% trypsin and 0.03% collagenase A for 25 min at 37 °C under gentle shaking. Cells were resuspended in neurobasal medium supplemented with 2% NS21 [56], 1 mM L-glutamine, 50 ng/mL NGF, 1% HEPES, and 5% FBS, and plated onto poly-D-lysine (PDL) (50 µg/mL) pre-coated 35-mm Petri dishes (Corning, NY, USA) at a density of 1000 cells/dish for patch clamp experiments after 6 h culture in vitro.

### 4.4. Patch Clamp Recording in DRG Neurons

Sodium currents in rat DRG neurons were recorded using a whole-cell patch clamp with an EPC-10 amplifier (HEKA Electronics, Germany) as described previously [57]. It has been reported that medium- (30–50 µm) to large-diameter (>50 µm) DRG neurons mainly express TTX-S VGSCs, while small-diameter (<30 µM) DRG neurons express both TTX-S and TTX-R VGSCs [30,58]. We therefore recorded TTX-S Na^+^ currents in medium- and large-diameter DRG neurons. Small-diameter DRG neurons were chosen to record TTX-R Na^+^ currents with bath application of 300 nM TTX. Current patch was recorded in small- and medium-diameter DRG neurons.

For voltage-clamp recordings, the extracellular solution was (in mM): NaCl 140, KCl 3, CaCl_2_ 1, MgCl_2_ 1, and HEPES 10 (pH adjusted to 7.4 with NaOH). The intracellular solution was (in mM): CsF 140, EGTA 1.1, NaCl 10, and HEPES 10 (pH adjusted to 7.3 with CsOH). Pipettes (1–3 MΩ) were pulled from 1.5-mm capillary tubing and filled with intracellular solution. For current clamp recordings, the intracellular solution contained (in mM): KCl 140, MgCl_2_ 5, CaCl_2_ 2.5, EGTA 5, ATP 4, GTP 0.3, and HEPES 10 (pH adjusted to 7.3 with KOH). The external solution contained (in mM): NaCl 140, MgCl_2_ 1, KCl 5, CaCl_2_ 2, glucose 10, and HEPES 10 (pH adjusted to 7.3 with NaOH). Voltage errors were minimized using 80% series resistance compensation, and the capacitance artifact was cancelled using computer-controlled circuitry of a patch clamp amplifier. Patchmaster (HEKA Electronics, Lambrecht/Pfalz, Germany, 2016) and OriginPro (Version 8, OriginLab, Northampton, MA, USA, 2018) were used to collect and analyze the experimental data, respectively. All data were presented as mean ± SEM, and each independent experimental cell was counted as an n number.

Concentration–response curves were fitted using a nonlinear logistic equation using prism software (Version 5.0, GraphPad Software Inc., San Diego, CA, USA, 2010). To study the effect of DHCT (30 μM) on current–voltage (I-V) relationships, the Na^+^ currents were triggered by depolarized pulses from −100 mV to +50 mV in a 5-mV step in the absence and presence of DHCT. The peak current recorded after each voltage step was normalized into conductance (G) according to the equation: I = G (V − V_rev_), where V_rev_ is the reversal potential of the sodium current. A steady-state activation curve was fitted using the Boltzmann equation: G/G_max_=1/(1 + exp(V_1/2_ − V)/*k*), where V is the membrane potential of the conditioning step, V_1/2_ is the membrane potential at half-maximal activation, and *k* is the slope factor. To study voltage dependent steady-state inactivation, Na^+^ currents were evoked by a 35-ms depolarizing pulse of 0 mV from −100 mV holding potential and pre-pulse ranging from −120 mV to 0 mV in a 10-mV step (100 ms). A steady-state inactivation curve was fitted using the Boltzmann equation: I/I_max_=1/(1 + exp(V_1/2_ − V)/*k*), where V is the membrane potential of the conditioning step, V_1/2_ is the membrane potential at half-maximal inactivation, and *k* is the slope factor. To quantify the affinity toward resting state or inactivating state, cells were held at −120 mV for 950 ms or −60 mV for 950 ms, then depolarized to 0 mV for 50 ms to induce Na^+^ current. To study the recovery time constant from fast and slow inactivation of VGSCs, DRG neurons were held at a holding potential of 0 mV for 100 ms or 10 s, followed by a variable recovery interval to −120 mV before testing for active current with a 20-ms pulse to 0 mV. The normalized amount of current measured was plotted versus the recovery duration to determine the τ value of recovery from inactivated state using first-, or second-order exponential growth equations as follows: y = y_0_ + A_1_e^x/t1^ and y = y_0_ + A_1_e^x/t1^ + A_2_e^x/t2^, respectively. For both equations y_0_ = the initial value at time 0, A_1_ = weight factor (of total recovered) for fraction recovered with time constant t_1_, A_2_ = weight factor (of total recovered) for fraction recovered at t_2_, x = the growth constant, t_1_ = time constant for recovery of fast inactivated channels, and t_2_ = time constant for recovery of potentially slow inactivated channels [34].

### 4.5. Chronic Constrictive Injury (CCI) Neuropathic Pain Model

Male rats (SD, 200–220 g) were randomly divided into 5 groups with 9 rats in each group. After anesthetized with 1% sodium pentobarbital (40 mg/kg), surgery was applied and sciatic nerves were exposed. Approximately one-fifth to one-fourth of the nerve was tied off. The sham group experienced surgical nerve exposing, but not ligating. After 14 days, animals were tested for development of mechanical allodynia by being placed on a wire mesh (20 × 15 × 20 cm, L × W × H) platform. After acclimatization for 15 min, a mechanical baseline was determined by applying a series of calibrated Von Frey fibers perpendicularly to the plantar surface of the hind paw of each rat. Positive responses included an abrupt withdrawal of the hind paw from the stimulus [59]. After a positive response was noted, a weaker fiber was applied. This was repeated until a 50% threshold for withdrawal could be observed.

### 4.6. Data Analysis

All data points were presented as mean ± SEM. The concentration–response curves were generated using GraphPad Prism 5.0 software using a non-linear logistic equation. Statistical significance between groups was calculated using T-test (two groups) or one-way ANOVA analysis when multiple groups were analyzed. *p* values below 0.05 were considered to be statistically significant.

## Supplementary Materials

The following are available online at https://www.mdpi.com/2072-6651/11/4/229/s1, Figure S1: TTX suppressed TTX-S Na^+^ currents in medium- and large-diameter DRG neurons.
