# Supplementary material for: Dehydrocrenatidine Inhibits Voltage-Gated Sodium Channels and Ameliorates Mechanic Allodia in a Rat Model of Neuropathic Pain
Source: Toxins (Basel). 2019 Apr 18;11(4):229. doi: 10.3390/toxins11040229 (PMC6521113; doi:10.3390/toxins11040229)
Supplement: Supplementary file 1 [file toxins-11-00229-s001.zip › toxins-482581-SI.pdf]

Supplementary Materials: Dehydrocrenatidine Inhibits Voltage-Gated Sodium Channels and Ameliorates Mechanic Allodia in a Rat Model of Neuropathic Pain

Fang Zhao, Qinglian Tang, Jian Xu, Shuangyan Wang, Shaoheng Li, Xiaohan Zou * and Zhengyu Cao *

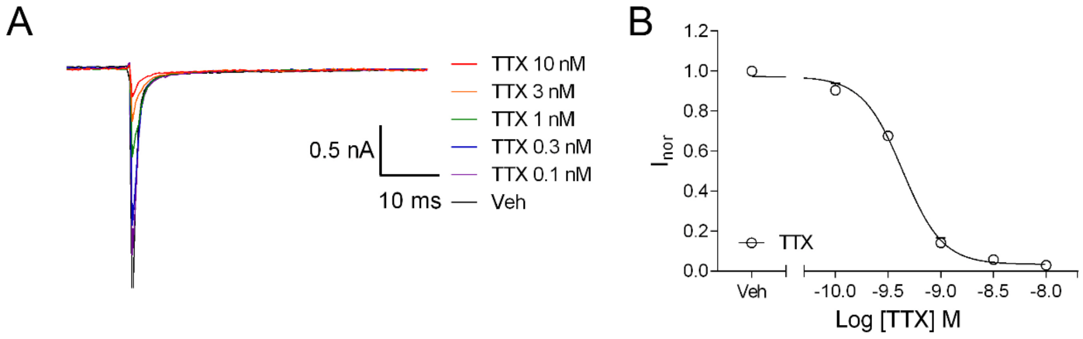

Figure S1. TTX suppressed TTX-S Na^+ currents in medium- and large-diameter DRG neurons. (**A**) Representative traces for TTX suppressing TTX-S Na^+ currents. (**B**) Concentration–response curve of TTX suppressed TTX-S Na^+ currents. Sodium currents were evoked by a 50 ms depolarization pulse to 0 mV from a holding potential of −100 mV. Each data point represents mean ± SEM (*n* = 5).
